# Mr. Duffin's New Shield Pessary

**Published:** 1831

**Authors:** 


### LXXII.

### Mr. Duffin's new Shield Pessary.

Mr. Duffin has invented a pessary, which he thinks superior to those in common use. It is a modification of the stalk pessary, and by means of a graduating screw, the length of the stalk can be accommodated to that of the vagina as the cure advances. The head, which is hollowed at its summit into a cup, being of a size adapted to that of the vagina in the particular case, and hollowed in proportion to the magnitude of the parts it has to receive, affords support without irritation or injury. The head may be made to unscrew, so that as the vagina contracts a smaller one may be substituted for it. The shield being pressed externally against the perinæum and perinæal part of the labia, and retained firmly in that situation by a thick T bandage of cotton, to which it may be attached, supports steadily through the medium of the head and stalk the weight of the prolapsed organ, and obviates all vacillation of the extremity of the stalk. Between the shield and stalk is a ball and socket-joint, allowing the instrument to accommodate itself to every change of posture, preventing any jarring impulse or undue friction. The pessary being hollow, and open at the extremity of the stalk, the shield also being perforated in this situation, whilst the head of the instrument also being perforated in numerous places, any astringent or other lotion can be thrown in by means of a syringe or India-rubber bottle. The instrument has received the approbation of Drs. Charles Clarke, Blundell, Henry Davies, and Robert Lee. The pessary is best made of ivory, but for economy the head and shield may be constructed of box-wood, the stem and joint of ivory. The instrument may be procured from Messrs. Stoddart, No. 401, Strand. A woodcut representing it is to be seen in the Medical Gazette for March 26th, 1831.

---

